# Links Between *N* ^6^-Methyladenosine and Tumor Microenvironments in Colorectal Cancer

**DOI:** 10.3389/fcell.2022.807129

**Published:** 2022-02-10

**Authors:** Yundi Zhang, Ke Zhang, Haoming Gong, Qin Li, Lajie Man, Qingchang Jin, Lin Zhang, Song Li

**Affiliations:** ^1^National Cancer Center/Cancer Hospital, Chinese Academy of Medical Sciences and Peking Union Medical College, Beijing, China; ^2^ Department of General Practice, Qilu Hospital, Cheeloo College of Medicine, Shandong University, Jinan, China; ^3^ Department of Physiology, School of Basic Medical Sciences, Cheeloo College of Medicine, Shandong University, Jinan, China; ^4^ Department of General Surgery, Qilu Hospital, Cheeloo College of Medicine, Shandong University, Jinan, China; ^5^ Department of Radiation Oncology, Qilu Hospital, Cheeloo College of Medicine, Shandong University, Jinan, China; ^6^ Department of Medical Oncology, Qilu Hospital, Cheeloo College of Medicine, Shandong University, Jinan, China

**Keywords:** colorectal cancer, immunotherapy, *N* ^*6*^-methyladenosine, tumor microenvironments, molecular classification

## Abstract

*N* ^6^-methyladenosine (m^6^A) is a critical epigenetic modification for tumor malignancies, but its role in regulating the tumor microenvironments (TMEs) has not been fully studied. By integrating multiple data sets and multi-omics data, we comprehensively evaluated the m^6^A “writers,” “erasers,” and “readers” in colorectal cancer and their association with TME characteristics. The m^6^A regulator genes showed specific patterns in co-mutation, copy number variation, and expression. Based on the transcriptomic data of the m^6^A regulators and their correlated genes, two types of subtyping systems, m^6^A_reg_Cluster and m^6^A_sig_Cluster, were developed. The clusters were distinct in pathways (metabolism/inflammation/extracellular matrix and interaction), immune phenotypes (immune-excluded/immune-inflamed/immune-suppressive), TME cell composition (lack immune and stromal cells/activated immune cells/stromal and immune-suppressive cells), stroma activities, and survival outcomes. We also established an m^6^Ascore associated with molecular subgroups, microsatellite instability, DNA repair status, mutation burdens, and survival and predicted immunotherapy outcomes. In conclusion, our work revealed a close association between m^6^A modification and TME formation. Evaluating m^6^A in cancer has helped us comprehend the TME status, and targeting m^6^A in tumor cells might help modulate the TME and improve tumor therapy and immunotherapy.

## Introduction

Colorectal cancer (CRC) is a major cause of cancer-related death worldwide (Siegel et al., 2020). Limited by treatment strategies, late-stage CRC has a 5-year survival rate of approximately 10% (Kuipers et al., 2015). In recent years, the therapeutic targets shifted from tumor cells to the tumor microenvironment (TME), consisting of a heterogeneous complex of immune cells, stromal cells, and extracellular matrix (Joyce and Pollard, 2009; Quail and Joyce, 2013). The anti-TME strategies, such as anti-angiogenetic drugs, immune checkpoint inhibitors (ICIs), and their combinations (*e.g.*, ICI plus angiogenesis or chemotherapy), were beneficial to only a part of patients (Tapia Rico and Price, 2018; Eng et al., 2019; Bourhis et al., 2021). It is essential to understand and evaluate the composition and activities of TMEs to guide clinical practice when using these treatments. A case in point in CRC is the immunoscore, which is calculated based on the TME cells and helps predict responses to chemotherapy or ICIs (Angell et al., 2020; Bruni et al., 2020).

*N* ^6^-Methyladenosine (m^6^A) is the most frequent epigenetic modification of RNA in eukaryotic cells (Frye et al., 2018). This process was reversibly regulated by its “writers,” “erasers,” and “readers.” It has multifaceted effects in deciding RNA fates, such as RNA transcription, splicing, structure, and translation, and participates in almost all physiological and pathological bioprocesses, including cancer development (Gaikwad et al., 2020). A connection between the m^6^A and TME is also present in some cancers. Based on multi-omics data, two studies evaluated the landscape of m^6^A modulators and found they were associated with immune cell infiltration in the TME and efficacies of ICIs in gastric cancer and renal carcinoma (Zhang et al., 2020a; Zhong et al., 2021). Recently, a specific study focusing on “writers” of four types of RNA modification and their relationship with immunotherapy efficacy was conducted in CRC (Chen et al., 2021a). However, a comprehensive study of three kinds of m^6^A regulators, including “writers,” “erasers,” and “readers,” in CRC has not been reported.

In the present study, we integrated the multi-omics and clinical data of seven CRC cohorts to evaluate the m^6^A modification patterns, TME characteristics, and their associations.

## Materials and Methods

### Data Sets

**FIGURE 1:**
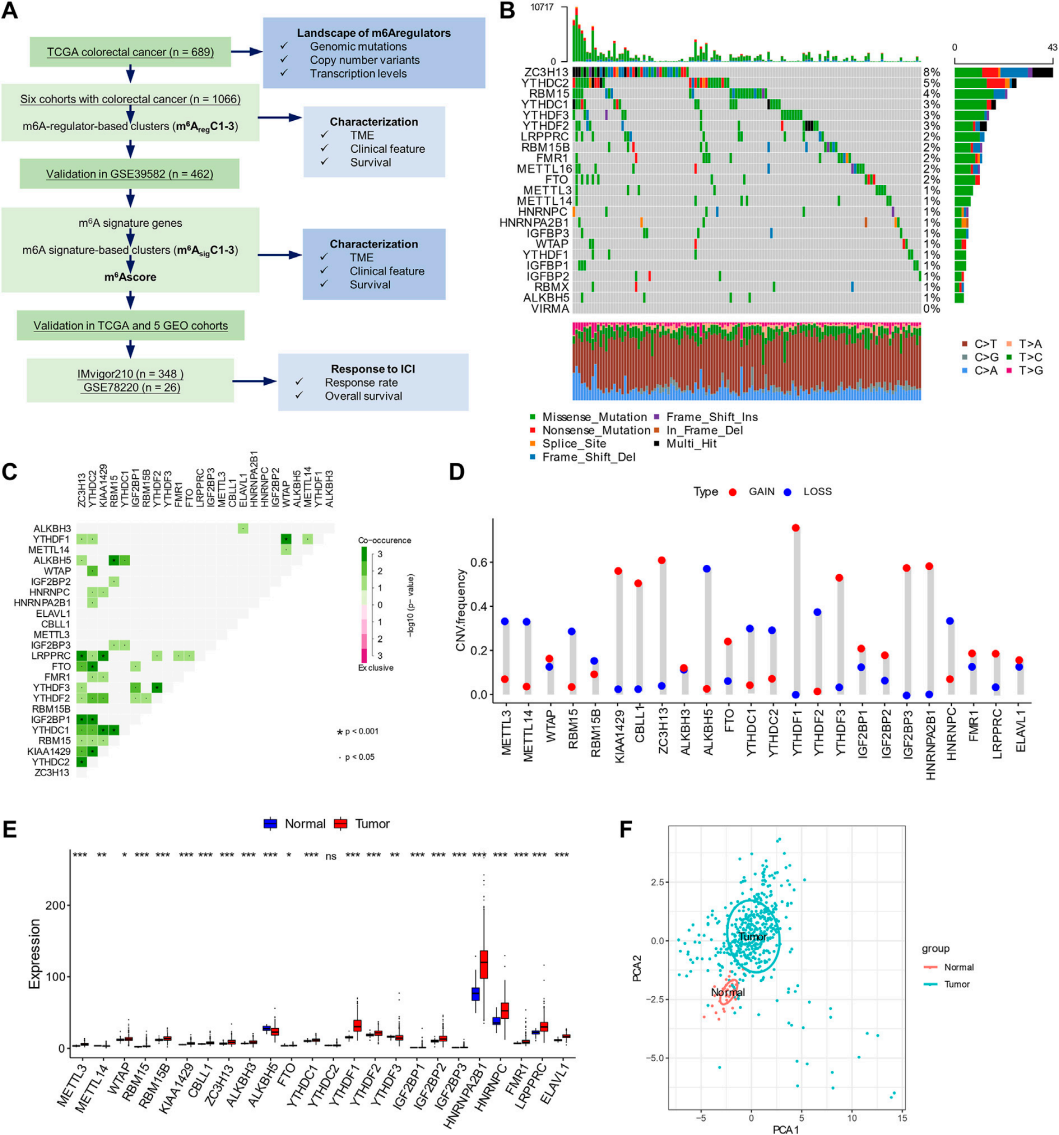
Workflow and landscapes of m^6^A regulators. **(A)** Workflow chart of this study with the main process. Cohorts used in this study are underlined. **(B)** Mutation rates of m^6^A regulators in The Cancer Genome Atlas (TCGA) data set. **(C)** Mutation co-occurrence analysis of m^6^A regulators in the TCGA data set. Co-occurrences with statistical significance (*p* < 0.05 and <0.001) are shown. **(D)** Copy number variants in the TCGA data set. **(E)** Expression levels of m^6^A regulators in normal and tumor tissues. **(F)** Principal component analysis for RNA level of 24 m^6^A regulators in the TCGA data set. *p < 0.05; **p < 0.01; ***p < 0.001.

Level 3 data from The Cancer Genome Atlas (TCGA), including expression, mutation, copy number variations, and clinical annotation, were downloaded from the TCGA database (https://tcga-data.nci.nih.gov/tcga/). The expression data and clinical information from six CRC cohorts (GSE17536, GSE29621, GSE33113, GSE37892, GSE38832, and GSE39582) were downloaded from the Gene Expression Omnibus (GEO) database (https://www.ncbi.nlm.nih.gov/geo). The GEO data were merged by R package “dplyr” and batch normalized by R package “sva.” The data from the two cohorts with ICI treatment, IMvigor210 and GSE78220, were obtained from the IMvigor210CoreBiologies package and GEO website, respectively. The study design and workflow are outlined in Figure 1A.

### Clustering According to *N* ^6^-Methyladenosine Regulators

The gene expression data of m^6^A regulators, including eight “writers” (*METTL3*, *METTL14*, *RBM15*, *RBM15B*, *WTAP*, *KIAA1429*, *CBLL1*, and *ZC3H13*), three “erasers” (*ALKBH3*, *ALKBH5*, and *FTO*), and 13 “readers” (*YTHDC1*, *YTHDC2*, *YTHDF1*, *YTHDF2*, *YTHDF3*, *IGF2BP1*, *IGF2BP2*, *IGF2BP3*, *HNRNPA2B1*, *HNRNPC*, *FMR1*, *LRPPRC*, and *ELAVL1*) were used for unsupervised clustering analysis. Cluster number determination and the following clustering were performed using the R package “ConsensusClusterPlus,” with 1000 times repetition. This method was used for clustering of m^6^A_reg_Clusters in the meta-GEO cohort, single GEO cohorts, and the TCGA cohort.

### Enrichment Analysis

Single-sample gene-set enrichment analysis and gene set variation analysis (GSVA) were used to quantify cell composition, immune checkpoints, CD8^+^ T-effector signature, epithelial–mesenchymal transition (EMT), angiogenesis, pan-fibroblast TGF² response signature (Pan-F-TBRS), WNT targets, DNA damage repair, mismatch repair, nucleotide excision repair, DNA replication, and antigen processing and presentation. The gene sets were derived from previous studies (Rosenberg et al., 2016; Şenbabaoğlu et al., 2016; Charoentong et al., 2017; Mariathasan et al., 2018) and have been summarized in a previous paper (Zhang et al., 2020a). The gene signatures of KEGG analysis were downloaded from the Molecular Signatures Database (http://www.gsea-msigdb.org/gsea/msigdb). The R package “gsea” was used.

### *N* ^6^-Methyladenosine Gene Signatures and m^6^A_sig_Clusters

The differentially expressed genes (DEGs) were identified by pairwise comparisons of three m^6^A_reg_Clusters by the “limma” R package. The overlapped genes among them were defined as m^6^A gene signatures. Tumors were unsupervised and clustered into three m^6^A_sig_Clusters by the R package “ConsensusClusterPlus” according to the expression levels of the m^6^A signature genes.

### Immune Cell Estimation

An abundance of 22 types of infiltrated immune cells were estimated by the software CIBERSORT (Newman et al., 2015) from the transcriptome data of CRC cohorts.

### Generation of m^6^Ascore

The m^6^Ascore was developed as follows: first, univariate Cox regression was performed for each m^6^A signature gene. Second, the dimensionality of the significant genes was reduced to two by principal component analysis (PCA) using the prcomp function in R. Third, PCA1 and PCA2 were summed up to get the m^6^Ascore for each patient.

### Survival Analysis

Survival outcomes were compared by log-rank regression and univariable COX regression. Confounding factors of survival prognosis were analyzed by multivariable COX regression. The Kaplan–Meier method and log-rank tests were performed by the R package “survminer.” The function “surv-cutpoint” was used for the determination of cut-off values in the cohorts.

### Statistical Analysis

The categorical variables were compared by Chi-square or Fisher’s exact tests. The continuous variables between the two groups were compared by t-test. The continuous variables among multiple groups were compared by one-way ANOVA or Kruskal–Wallis tests. The Benjamini–Hochberg methods were used to correct *p*-values for multiple testing. The survival distributions were compared by log-rank regression and COX regression. Correlations were calculated by linear regressions. The data were analyzed with the R (version 3.6.3) and R Bioconductor packages. A *p*-value < 0.05 was considered statistically significant.

## Results

### Landscape of *N* ^6^-Methyladenosine Regulator Gene Mutation, Copy Number, and Expression in Colorectal Cancer

According to previous reports, a total of 24 m^6^A regulators, including eight “writers” (*METTL3*, *METTL14*, *WTAP*, *RBM15*, *RBM15B*, *KIAA1429*, *CBLL1*, and *ZC3H13*), three “erasers” (*ALKBH3*, *ALKBH5*, and *FTO*), and 13 “readers” (*YTHDC1-2*, *YTHDF1-3*, *IGF2BP1-3*, *HNRNPA2B1*, *HNRNPC*, *FMR1*, *LRPPRC*, and *ELAVL1*) were included for analysis in this study (Figure 1B). A frequency of 24.11% had at least one mutation on the m^6^A regulators. The “readers” such as *ZC3H13*, *YTHDC2*, *YTHDC1*, *YTHDF3*, and *YTHDF2* were the most frequently mutated genes, while most “writers” (except *RBM15*) and “erasers” were less mutated (Figure 1B). High percentages of mutation co-occurrences between 11 pairs of genes were detected (*p* < 0.001; Figure 1C). Most of these were “reader–writer” and “reader–eraser” co-mutations (Figure 1C). No mutation co-occurrence between “writers” or “erasers” was found (Figure 1C).

Copy number variations were significant in some m^6^A regulators (Figure 1D, Supplementary Figure S1). Changes of *YTHDF1/3*, *HNRNPA2B1*, *IGF2BP2/3*, *CBLL1*, *KIAA1429*, *ZC3H13*, and *FTO* were dominantly gains, while those of *YTHDF2*, *ALKBH5*, *RBM15*, *METTL14*, *YTHDC1*, *HNRNPC*, *METTL3*, and *YTHDC2* were dominantly losses (Figure 1D).

The RNA levels of most m^6^A regulators were significantly different between normal and tumor samples, with 22 genes upregulated and ALKBH5 downregulated in tumor tissues (Figure 1E). The PCA of RNA expression distinctly distinguished tumor from normal samples (Figure 1F).

### Clustering Colorectal Cancer by *N* ^6^-Methyladenosine Regulators

**FIGURE 2:**
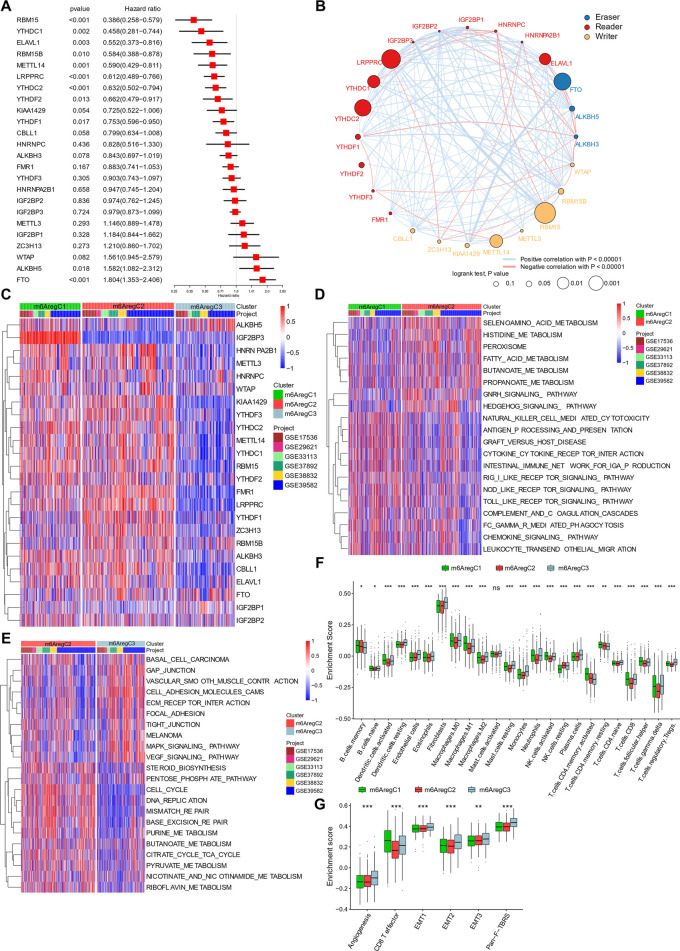
Clustering of m^6^A regulator–based subtypes in meta-data of six Gene Expression Omnibus cohorts. **(A)** Hazard ratio of m^6^A regulators in predicting survivals in CRC patients. **(B)** Interaction among m^6^A regulators in colorectal cancer. Line colors represent positive or negative correlation, and thickness represents correlation strength. Colored circles indicate the types of m^6^A regulators, and circle sizes indicate prognostic ability. **(C)** Unsupervised clustering based on 24 m^6^A regulators. Three clusters, termed m^6^A_reg_C1–3, were defined. **(D–E)** Differential biological pathways between m^6^A regulator–based clusters. The pathways were quantified by gene set variation analysis enrichment and compared between C1 and C2 **(D)** and C2 and C3 **(E)**. **(F)** Abundance of tumor-infiltrating cells in three subtypes. **(G)** Enrichment of stroma-activated pathways in three subtypes. One-way ANOVA tests compared the three groups in **(F**,**G)**. *p < 0.05; **p < 0.01; ***p < 0.001.

A total of six GEO data sets (GSE17536, GSE29621, GSE33113, GSE37892, GSE38832, and GSE39582), including 1066 CRC patients, were pooled for survival analysis. About 11 of the 24 m^6^A regulators had prognostic roles in patients by univariate Cox regression (Figure 2A). Among them, the “erasers” ALKBH5 and FTO had a significantly high hazard ratio of death, while nine “readers” and “writers” were associated with better survival (Figure 2A).

Based on prognostic values of m^6^A regulator RNA levels and their intercorrelations, a correlation network was constructed (Figure 2B). Positive correlations were prevalent among m^6^A regulators. The highest correlations were found between RBM15B and IGF2BP3, KIAA1429 and FTO, and YTHDC2 and IGF2BP1 (Figure 2B). Negative correlations also occurred among the three groups (Figure 2B). These indicated a cross talk between the m^6^A regulators.

Under unsupervised clustering, the patients were classified into three subgroups with different m^6^A regulator expression patterns, named m^6^A regulator–based Cluster 1–3 (m^6^A_Reg_C1-3) (Figure 2C and Supplementary Figure S2). C1 was characterized with high expression of IGF2BP3, and C3 was characterized with overexpression of ALKBH5 and FTO and downregulation of the other regulators (Figure 2C, Supplementary Figure S3). C2 was characterized by the low expression of ALKBH5, and high levels of some readers, including FMR1, LRPPRC, HNRNPA2B1, and YTHDF1 and 3 (Figure 2C, Supplementary Figure S3). The three clusters showed different survivals, C1 and C2 showing better outcomes than C3 (Supplementary Figure S4).

### *N* ^6^-Methyladenosine Regulator–Based Subtypes Are Different in Tumor Microenvironment Composition

Activation of pathways within the three m^6^A regulator–based subtypes was analyzed by GSVA. Comparing C1 and C2, C1 was characterized by the inflammation pathways, including pattern recognition (RIG I, NOD-like, and Toll-like receptor pathways), cytotoxicity (NK cell–mediated cytotoxicity and FCγ-mediated phagocytosis), and chemokines (chemokine signaling pathway, cytokine–cytokine receptor interaction, and leukocyte transendothelial migration; Figure 2D), while C2 was characterized by metabolism (selenoamino acid metabolism, histidine metabolism, fatty acid metabolism, butanoate metabolism, and propanoate metabolism) (Figure 2D). When we compared cluster C2 and C3, C2 was still enriched in metabolic pathways (pentose phosphate pathway, purine metabolism, butanoate metabolism, citrate cycle–TCA cycle, pyruvate metabolism, and riboflavin metabolism), while C3 was characterized with cell–extracellular matrix and cell–cell connections (gap junction, focal adhesion, and tight junction; Figure 2E).

Due to the prominent differences in inflammation and ECM connections, we then used CIBERSORT to evaluate the TME composition in these subtypes. Like immune inflamed cancer, C1 showed activated DC cells, M1 macrophage, activated NK cells, activated CD4^+^ T memory cells, CD8^+^ cells, and follicular T helper cells (Figure 2F). C3 was highly infiltrated with stroma cells (endothelial cells, fibroblasts), resting cells (monocytes, M0 macrophages, resting DC cells, resting NK cells), and immune suppressive cells (M2 macrophages and regulatory T cells), representing an excluded immunity (Figure 2F). Further GSVA showed an enhanced stromal activity in C3, including signatures of angiogenesis, EMT 1–3, and pan-fibroblast TGFβ responses (Figure 2G). By contrast, C1 had the highest CD8^+^ T-effector signature (Figure 2G). C2 was likely immune-ignored cancer due to a lack of all types of immune and stromal cells (Figure 2G).

### *N* ^6^-Methyladenosine Regulator–Based Subtypes Are Related to Clinical Features

**FIGURE 3:**
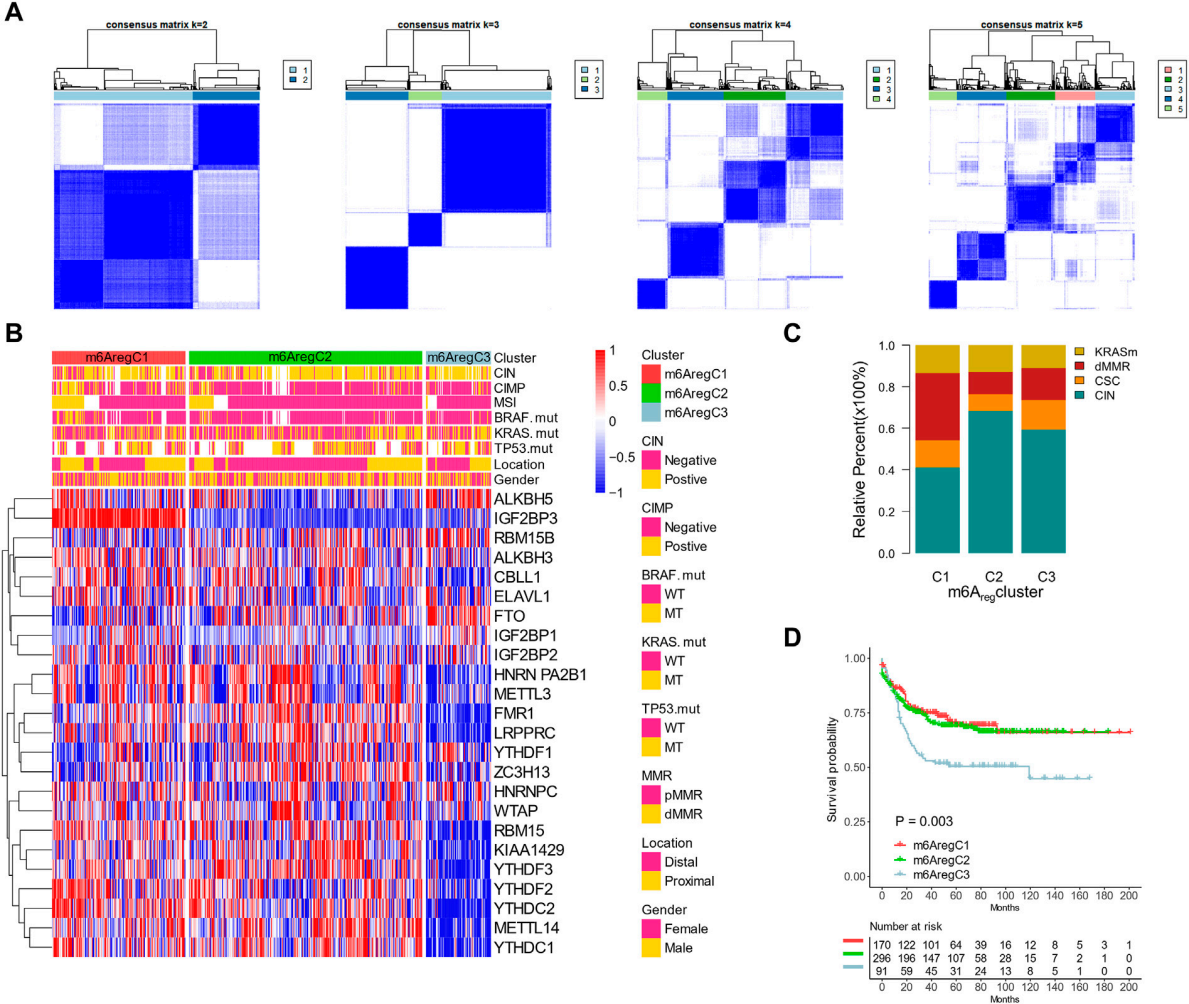
Association between m^6^A regulator–based subtypes and tumor microenvironment composition. **(A)** Unsupervised clustering based on m^6^A regulators with *n* = 2 to 5 in the GSE39582 data set. **(B)** Clustering m^6^A regulators into three subtypes. Distribution of molecular subtypes (chromosomal instability, CpG island methylator phenotype, and microsatellite instability) and driver mutations (KRAS, RBAF, and TP53) were provided. **(C)** Distribution of genetic change types in three m^6^A regulator–based subtypes. **(D)** Kaplan–Meier curves of the three m^6^A regulator–based subtypes.

To validate and further explore the clinical features of the three subtypes, we used the GSE39582 cohort with detailed clinical and molecular information for further analyses. Unsupervised clustering with m^6^A regulators showed an optimal reclassification of the three subgroups (Figures 3A,B). C1 had more CpG island methylator phenotype (CIMP) status (Figure 3C). C3 had less microsatellite instability (MSI) status and more chromosomal instability (CIN) status than the other two subgroups (Figure 3B). The mutation rates of BRAF, KRAS, and TP53 were similar among C1–3 (Figure 3B). With another molecular subtype system, the Cartes d’Identité des Tumeurs classification system, C1 patients were characterized with more dMMR and fewer CIN patients, while C2 had the most CIN subtypes (Figure 3C). In addition, Kaplan–Meier revealed survival differences among the three subtypes, with m^6^A_reg_C3 with an inferior prognosis (Figure 3D). The validation was also performed on TCGA, which was also divided into three clusters with survival differences (Supplementary Figure S5A and Supplementary Figure S5B).

### Generation of *N* ^6^-Methyladenosine–Related Gene Signatures and Signature-Based Clusters

**FIGURE 4:**
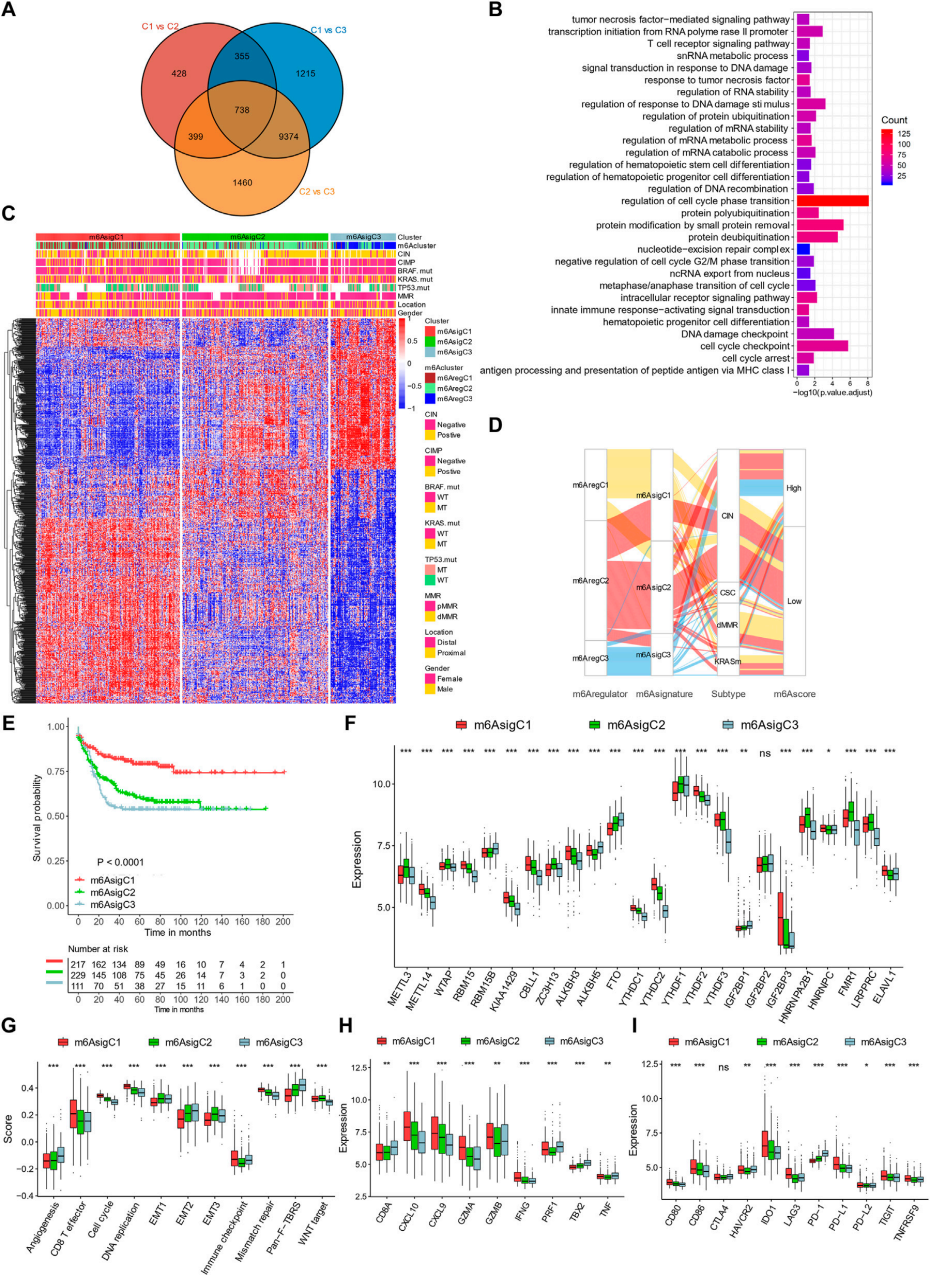
Construction of m^6^A signature–based clusters. **(A)** Overlaps of differential expression genes among the three m^6^A regulator–based subtypes. **(B)** Gene Ontology enrichment of the m^6^A signature genes. **(C)** Clustering patients based on m^6^A signature genes into three subtypes termed m^6^A_sig_C1–3. **(D)** Alluvial diagram connecting m^6^A_reg_Clusters, m^6^A_sig_Clusters, gene mutation subtypes, and m^6^Ascores. **(E)** Kaplan–Meier curves of the three m^6^A signature–based subtypes. **(F)** Expression levels of m^6^A regulators in three m^6^A signature–based subtypes. **(G–I)** Signatures of stromal activation **(G)**, immune activation **(H)**, and immune checkpoints **(I)** in three m^6^A signature–based subtypes. *p < 0.05; **p < 0.01; ***p < 0.001.

To define a gene signature related to the m^6^A regulators, we examined the DEGs among them. In total, 738 genes were shared among the DEGs by pairwise comparisons of three m^6^A_reg_Clusters, which were termed m^6^A-related gene signatures (Figure 4A). The signature genes were enriched in pathways related to RNA metabolism, validating the roles of the m^6^A regulators on RNA fates. They were also enriched in terms related to immunity (tumor necrosis factor, T-cell receptor signaling, innate immune responses, and antigen processing and presentation), DNA damage responses (signal transduction in response to DNA damage, regulation of responses to DNA damage stimulus, DNA recombination, nucleotide–excision repair complex, and DNA damage checkpoint), and cell cycle (e.g., cell cycle checkpoint, cell cycle arrest, and metaphase/anaphase transition of cell cycles; Figure 4B). These indicated that immunity, DNA damage responses, and cell cycles might be regulated by m^6^A modification.

To further evaluate this m^6^A regular–related signature, we performed further unsupervised clustering and got three m^6^A signature–based clusters (m^6^A_sig_C1–3; Figure 4C, Supplementary Figure S6). The three signature-based subgroups overlapped with the m^6^A regulator–based subgroups well (Figures 4C,D) and showed similar clinical features (Figures 4C,D). The m^6^A_sig_C1 showed superior survival outcomes than m^6^A_sig_C2 and C3 (Figure 4E). In addition, they had different expression levels of 23/24 m^6^A regulators (Figure 4F).

By evaluating pre-defined signatures, we found the m^6^A_sig_C1 was characterized with immune activation, with high CD8^+^ effector T cells (Figure 4G), transcripts of immune activation (Figure 4H), and immune checkpoints (Figure 4I). By contrast, C3 was characterized with stromal components, including angiogenesis and Pan-F-TBRS (Figure 4G).

### Generation of *N* ^6^-Methyladenosine Score and Its Predictive Ability of Tumor Microenvironment and Clinical Feature

**FIGURE 5:**
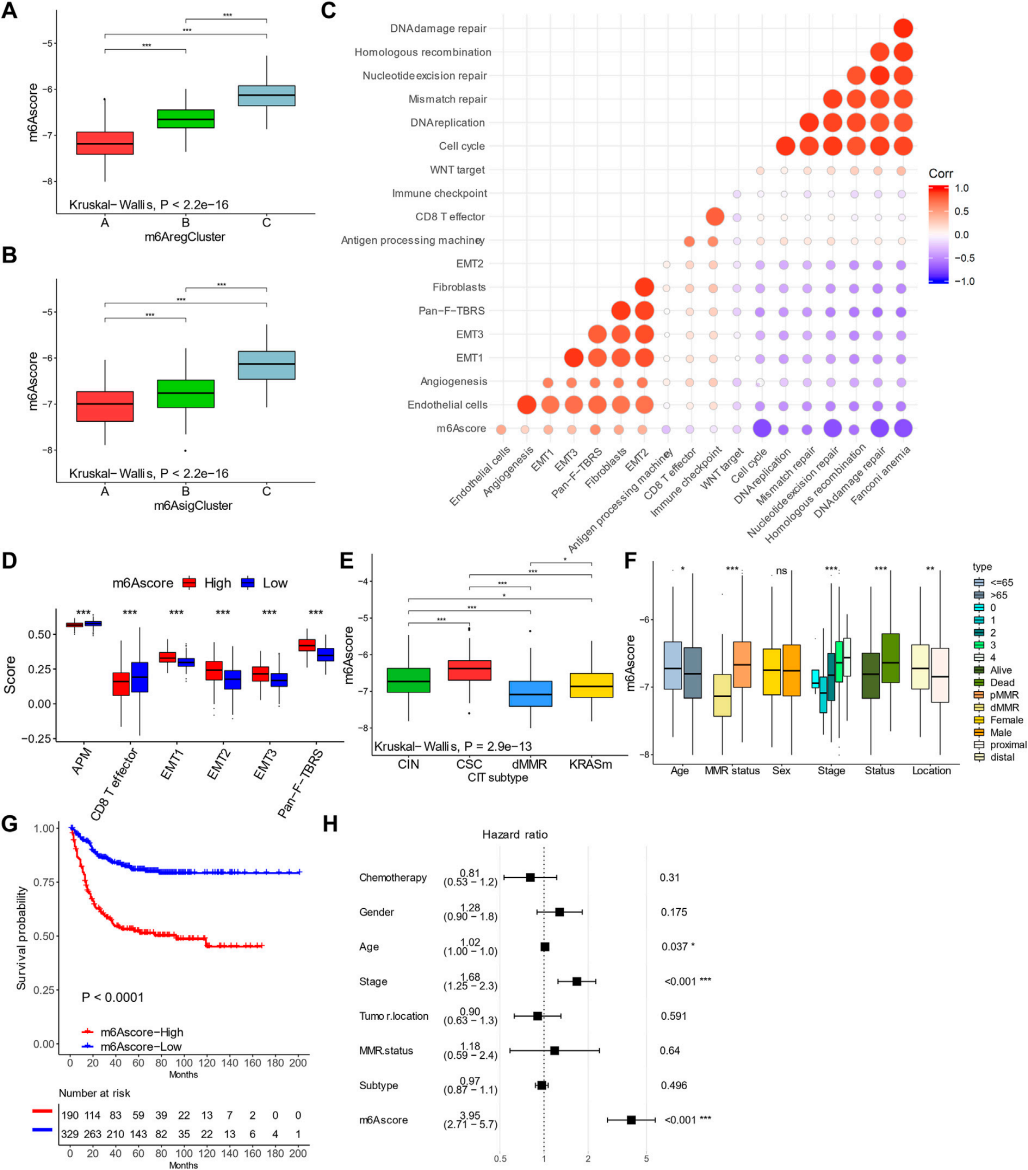
Characteristics of m^6^Ascore in colorectal cancer. **(A,B)** m^6^Ascores in m^6^A regulator–based **(A)** and signature–based **(B)** clusters. **(C)** Correlations between m^6^Ascores and gene signatures in colorectal cancer. **(D)** Levels of stromal activity in patients with high and low m^6^Ascores. **(E,F)** Distribution of m^6^Ascores in patients with different genomic change subtypes and clinical features. **(G)** Kaplan–Meier curves of patients with high and low m^6^Ascores. **(H)** Forest plot showing multivariable COX results of m^6^Ascore and clinical features in predicting death. *p < 0.05; **p < 0.01; ***p < 0.001.

To quantify m^6^A modification patterns, we defined an m^6^Ascore based on the m^6^A signature genes. The majority of m^6^A_reg_C1 and m^6^A_sig_C1 had a low m^6^Ascore, while patients with high scores were mainly m^6^A_reg_C2/3 or m^6^A_sig_C2/3 (Figure 4D). Correspondingly, m^6^A_reg_C1 and m^6^A_sig_C1 both showed a lower median m^6^Ascore than the other two groups (Figures 5A,B). The m^6^Ascore positively correlated with stromal signatures, including endothelial cells, angiogenesis, EMT 1/2/3, Pan-F-TBRS, and fibroblasts. We found an inverse correlation with signatures of immune activation (CD8^+^ T, antigen processing, immune checkpoints) and DNA damage responses (DNA replication, mismatch repair, nucleotide excision repair, homologous recombination, DNA damage repair, and Fanconi anemia), suggesting that a low m^6^Ascore was linked with immune activation, while a high m^6^Ascore was linked with stromal activation (Figure 5C). Consistent with this, patients with a high m^6^Ascore had a low CD8^+^ T score but enhanced activation of the stromal pathways (Figure 5D).

In addition, most dMMR patients had a low m^6^Ascore (Figure 4D) and a lower median m^6^Ascore than the other groups (Figure 5E). By contrast, the CSC-subtype patients had the highest m^6^Ascore (Figure 5E). The m^6^Ascore was also associated with many clinical features; younger patients (age <65 years), high AJCC stages, distal location, and pMMR were significantly associated with a higher m^6^Ascore (Figure 5F). By univariate analysis, patients with a low m^6^Ascore showed a remarkably superior survival than the m^6^Ascore-high group, with a hazard ratio of death of 0.2474 (95% CI, 0.172–0.3561) and *p*-value < 0.001 (Figure 5G). A multivariate cox regression model was also used to exclude the confounding factors for patients' survival, including chemotherapy, gender, age, stage, tumor location, MMR status, and molecular subtype (Figure 5H). The results also showed that m^6^Ascore is still an independent prognostic biomarker for evaluating patient outcomes, with a hazard ratio of death of 3.95 (95% CI, 2.71–5.70 and *p*-value < 0.001; Figure 5H).

### Validation of *N* ^6^-Methyladenosine Score in The Cancer Genome Atlas and Five Gene Expression Omnibus Data Sets

**FIGURE 6:**
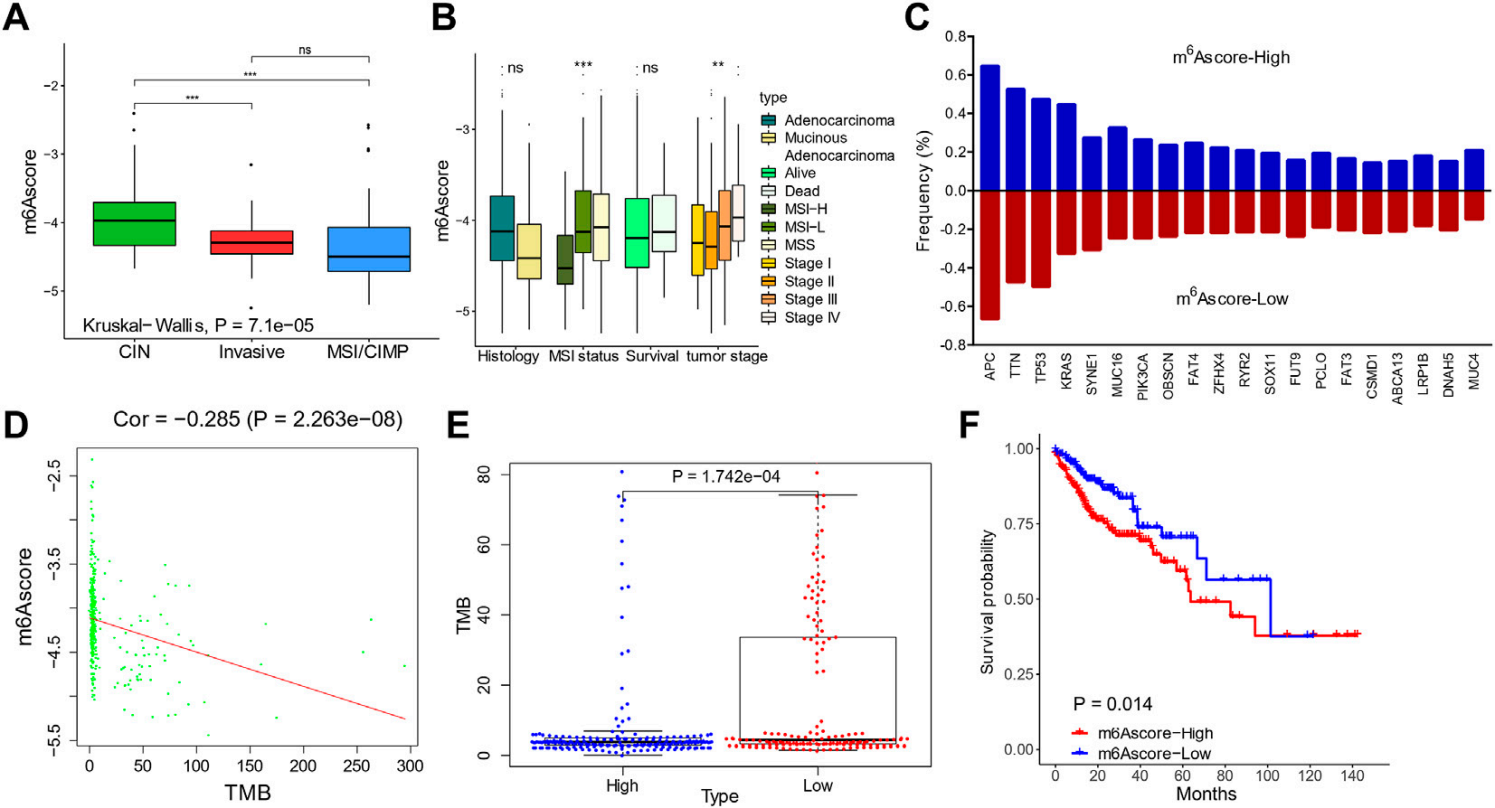
Validation of m^6^Ascores in The Cancer Genome Atlas (TCGA) cohorts. **(A,B)** m^6^Ascores in patients with different molecular subtypes **(A)** and clinical features **(B)**. **(C)** Genomic mutation rates of the top 20 genes in patients with high and low m^6^Ascores. **(D)** Correlation between m^6^Ascores and tumor mutation burdens. **(E)** Tumor mutation burdens in patients with high and low m^6^Ascores. **(F)** Kaplan–Meier curves of m^6^Ascores.

We then validated the prognostic value of m^6^Ascore in the TCGA data set. When stratifying patients by molecular subtypes, the MSI/CIMP patients showed the lowest m^6^Ascore, and CIN patients showed the highest m^6^Ascore (Figure 6A). The m^6^Ascore was also associated with the MSI status and tumor stages; the MSI-H and stage I/II patients had a low m^6^Ascore (Figure 6B).

The mutation landscapes were compared between low-m^6^Ascore and high-m^6^Ascore patients (Figure 6C). Frequencies of the top 20 mutations were similar, except of *KRAS*, which occurred more frequently in the m^6^Ascore-high patients (44.7 vs. 32.6%; Figure 6C). The m^6^Ascore and TMB were negatively correlated (Figure 6D), with higher TMB in low-m^6^Ascore tumors than in m^6^Ascore-high tumors (Figure 6E). Patients with a low m^6^Ascore also showed prolonged survival compared to patients with a high m^6^Ascore, with a hazard ratio of death of 0.5345 (95% CI, 0.3137–0.9109) and *p*-value 0.014 (Figure 6F).

**FIGURE 7:**
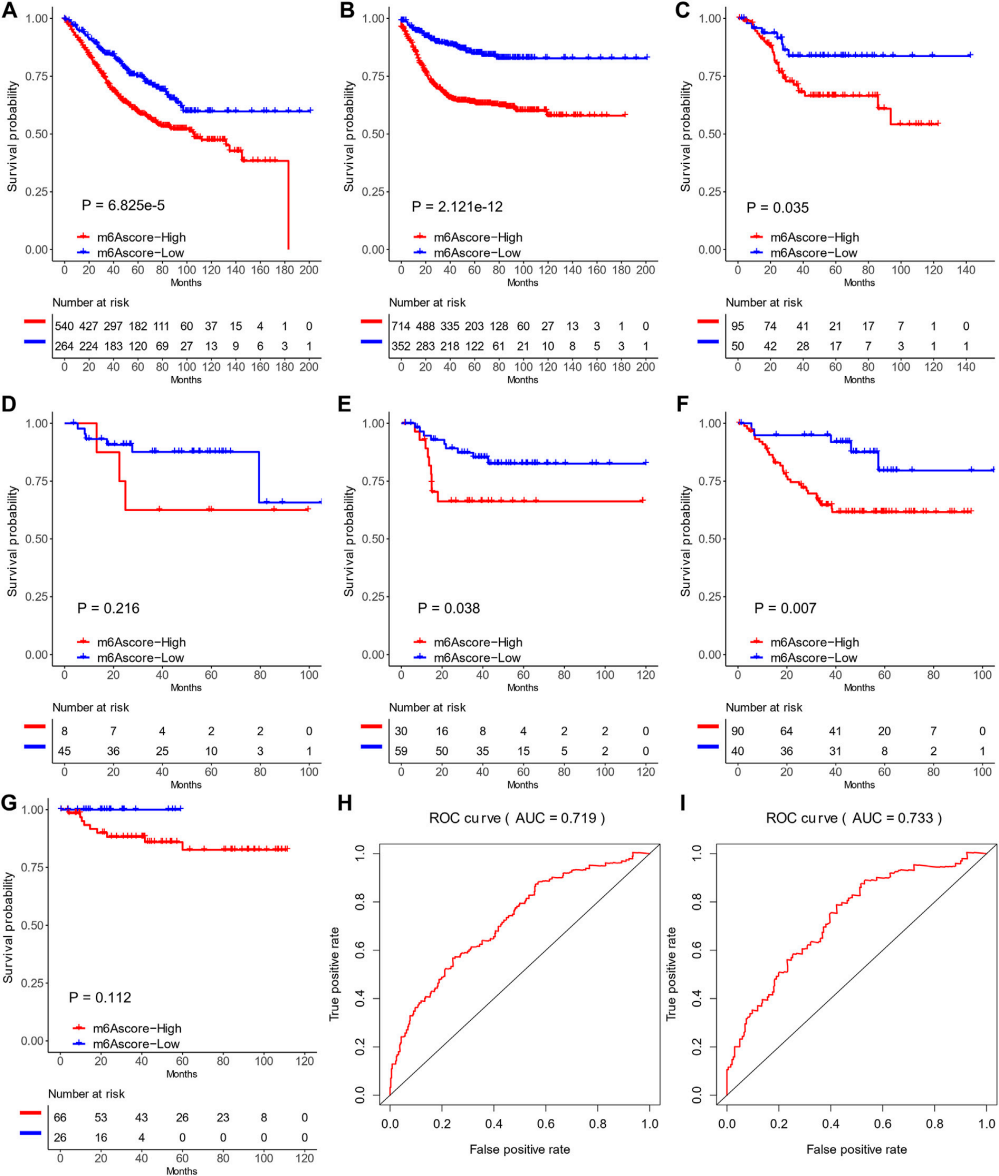
Prediction values of m^6^Ascores in six Gene Expression Omnibus data sets. **(A,B)** Recurrence-free survival **(A)** and overall survival **(B)** of patients with high and low m^6^Ascores in six data sets. **(C–G)** Kaplan–Meier curves of patients with high and low m^6^Ascores in GSE17536 **(C)**, GSE29621 **(D)**, GSE33113 **(E)**, GSE37892 **(F)**, and GSE38832 **(G)**. **(H,I)** ROC curves of recurrence-free survival **(H)** and overall survival **(I)** in six data sets.

We further evaluated the prognostic ability of the m^6^Ascore in TCGA and the other cohorts (GSE17536, GSE29621, GSE33113, GSE37892, and GSE38832; Figures 7A–G) to validate its stability. The low-m^6^Ascore was associated with more prolonged relapse-free survival (Figure 7A) and overall survival (Figure 7B) in the combined cohorts. The area under the curve to predict 3-year and 5-year survivals was 0.719 and 0.733, respectively (Figures 7H,I).

### Prediction of Immunotherapy Outcomes by *N* ^6^-Methyladenosine Score

**FIGURE 8:**
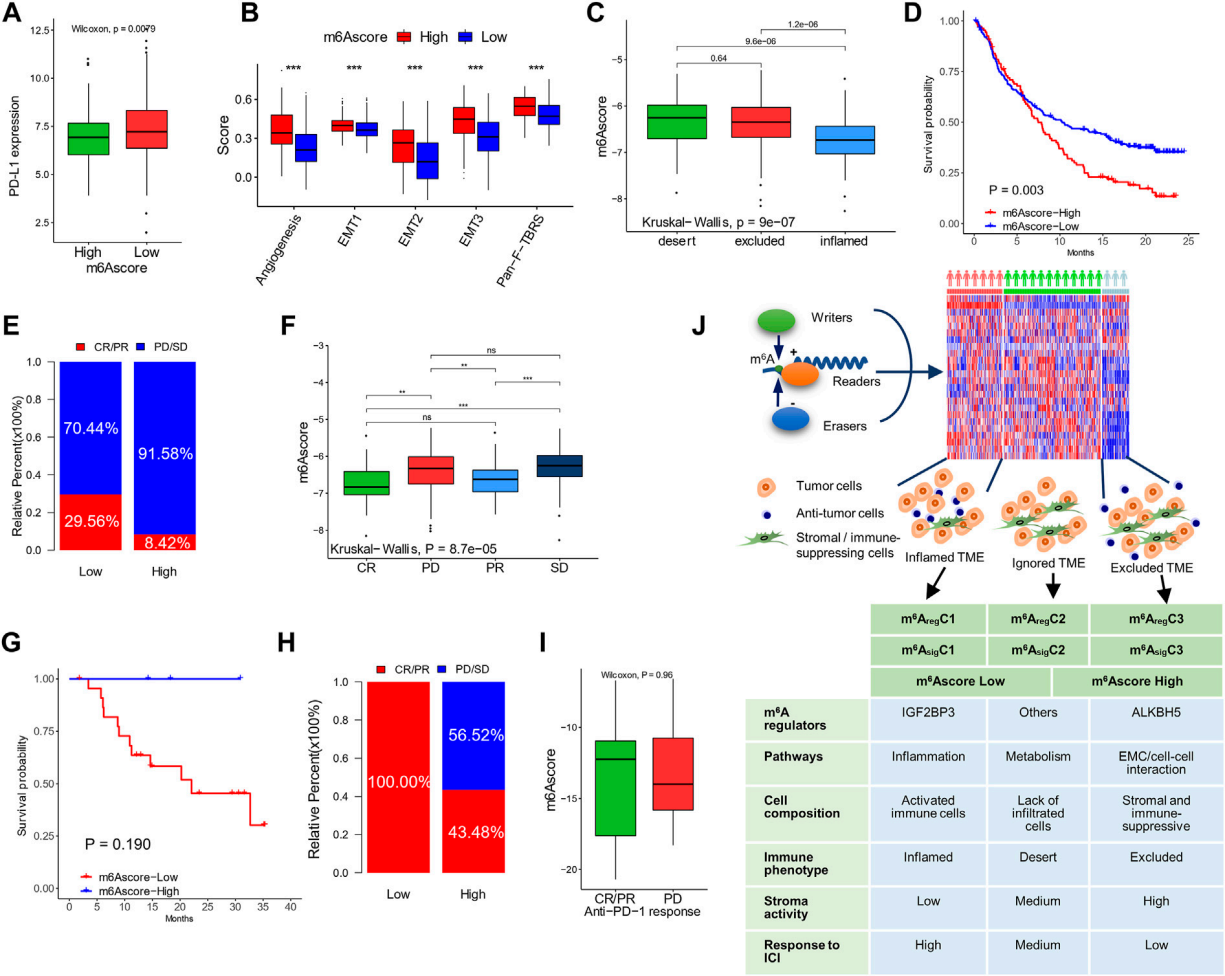
The ability of m^6^Ascore to predict responses to ICI. **(A)** PD-L1 expression in patients with high and low m^6^Ascores in the IMvigor210 cohort. **(B)** Stromal activation signatures in patients with high and low m^6^Ascores. **(C)** m^6^Ascores in the ignored, excluded, and inflamed types of tumors. **(D,E)** Kaplan–Meier curves **(D)** and response rates **(E)** in patients with high and low m^6^Ascores after treatment of ICI. **(F)** m^6^Ascores in patients with different responses to ICI. Data of **(A–F)** were from the IMvigor210 cohort. **(G,H)** Kaplan–Meier curves **(G)** and response rates **(H)** to ICI in patients with high and low m^6^Ascores after treatment of ICI in the GSE78220 cohort. **(I)** m^6^Ascores in patients with different responses to ICI in the GSE78220 cohort. **(J)** Graphic abstract of this study (top) and characteristics of the subtypes (bottom). *p < 0.05; **p < 0.01; ***p < 0.001.

Due to the close association between the m^6^A status and immunotherapy biomarkers (MSI, DDR, TMB, immune checkpoints, and stromal scores), we evaluated the ability of the m^6^Ascore to predict responses to ICIs, using two cohorts (IMvigor210 and GSE78220), with ICI treatment. The IMvigor210 cohort included 310 PD-L1–treated patients, who were classified into three immune subgroups, including “ignored,” “excluded,” and “inflamed” (Rosenberg et al., 2016). In accordance with the former study, patients with a low m^6^Ascore showed higher expression of PD-L1 (Figure 8A) and lower expression of stromal signatures (angiogenesis, EMT 1/2/3, and Pan-F-TBRS; Figure 8B) than patients with a high m^6^Ascore. The “inflamed” patients showed a significantly higher m^6^Ascore than the other two subtypes (Figure 8C). Clinically, patients with a low m^6^Ascore exhibited more prolonged survival (hazard ratio, 0.58; 95% CI, 0.40–0.83; *p*-value = 0.003; Figure 8D) and a higher response rate (29.56 vs. 8.42%, Figure 8E) than patients with a high m^6^Ascore. Correspondingly, the patients with complete and partial responses showed a significantly lower m^6^Ascore than patients with stable or progressing disease (Figure 8F). The prognostic value of the m^6^Ascore in ICI-treated patients was also validated in GSE78220, although the differences were not statistically significant due to limited sample sizes (Figures 8G–I).

## Discussion

m^6^A is a critical epigenetic mechanism for regulating tumor malignancies by promoting proliferation, migration, stemness, drug sensitivity, and resistance (Lan et al., 2021). Nonetheless, its role in TME regulation has been less studied. This needs a comprehensive analysis of both m^6^A and TME components simultaneously. In this study with multi-omics data, we revealed a specific pattern in co-mutation, copy number variation, and expression of m^6^A “writers”, “erasers”, and “readers” in the CRC samples. Molecular differences between colon and rectal cancers were not seen. In unsupervised clustering, two types of subtyping methods—m^6^A_reg_Cluster and m^6^A_sig_Cluster—were distinct in the pathways, TME cell composition, immune phenotypes, stroma activities, and survival outcomes (Figure 8J). Based on the m^6^A regulator–related signatures, we also established an m^6^Ascore associated with molecular subtyping, MSI and DNA repair status, tumor mutation burdens, survival, and responses to immunotherapy. These results indicated a close relationship between m^6^A modification and anti-tumor immunity in CRC, shedding light on a future direction to evaluate and modulate TME by targeting m^6^A.

This connection was not unique in CRC, since such phenomenon was also found in other types of cancers, such as gastric cancer (Zhang et al., 2020a). Pan-cancer analyses also showed that the m^6^A regulators, mainly “writers” and “erasers,” were differentially expressed in different TME subtypes (Zhu et al., 2020). Recently, a study focusing on “readers” of RNA modification and their relationship with TME was conducted in CRC (Chen et al., 2021a). Indeed, our study found a genetic pattern of m^6^A regulators, especially the “readers.” For example, “reader-writer” and “reader-eraser” co-mutations were frequent, while “writer-eraser” co-mutation was not found, suggesting an important role of “readers” in tumorigenesis and potential driving ability of “writer-reader” or “eraser-reader” communications. Consistent with this, many studies revealed that “writers” or “erasers” regulate tumor malignancies in a “reader”-dependent manner (Li et al., 2019). Copy numbers of two main “erasers,” *ALKBH5* (loss) and *FTO* (gain), were inversely related. Accordingly, their relative expression levels compared to normal tissue were also inversely related (ALKBH5 down and FTO up). The different targets and functions between them have been reported by previous studies (Wei et al., 2018). This imbalance of “erasers” might be another mechanism in CRC tumorigenesis and targeted by specific inhibitors, such as meclofenamic acid (Huang et al., 2015). In this study, we found ALKBH5 and FTO had parallel values in predicting outcomes of patients and were both highly expressed in m^6^AregC3, suggesting these two erasers cooperate in shaping the RNA modification patterns and impacting patients' survival.

Despite the genetic patterns that were different from normal tissues, heterogeneity of m^6^A regulator expressions was found among patients. By clustering with m^6^A regulators or m^6^A signature genes, three clusters were obtained. The heterogeneity has also been observed by other groups. For example, Ogino and Goel (2008), De Sousa E Melo et al. (2013), Sadanandam et al. (2013), Marisa et al. (2013), and Roepman et al. (2014) provided their classification systems to divide the CRC patients into three to six subtypes, yet being different in methodology, inclusion criteria, and interpretations (Singh et al., 2021). In 2015, a consensus of molecular subtypes of CRC was raised based on large patient cohorts and CRC was categorized into five subtypes (Guinney et al., 2015). Different from these previous subtyping methods, which mainly used mutation and epigenetics data (Singh et al., 2021), our subtyping method was based on the transcriptomic data of limited genes (22 or 738). Our method had a strong ability to predict survival outcomes, was reliable across multiple cohorts, and overlapped with other classification systems well. These findings suggest that subtyping by m^6^A regulators or m^6^A signatures was meaningful and clinically feasible.

In this era of immunotherapy, exploring immune TME is becoming a hot issue these days. The initial work on ICI in CRC showed limited success (Topalian et al., 2012). The following studies discriminate the dMMR/MSI-H patients with high responses to ICI (Chung et al., 2010; Overman et al., 2018). Combination with other therapeutic regimens, such as regorafenib, FOLFOX, or cetuximab, was also beneficial to a part of microsatellite stable (MSS) patients (Tapia Rico and Price, 2018; Eng et al., 2019; Bourhis et al., 2021). Therefore, subtyping CRC in the aspect of immune activity or TME is important for identifying “hot” tumors that may benefit from immunotherapy in MSS CRC. Becht et al. (2016) characterized the immune and stromal features of 1,388 CRC and found that they were highly correlated with the CRC subtypes. Our subgroups also have a potent ability to differentiate immune orientations. The m^6^A_reg_C1 and m^6^A_sig_C1 were likely “hot” tumors, characterized by the activation of inflammation pathways, the infiltration of active immune cells, and a lack of stromal components. This subtype represents an inflammatory type of cancer that responds well to immunotherapy. The m^6^A_reg_C3 and m^6^A_sig_C3 were characterized by high stroma activity, immune-suppressive cells, and resting immune cells, which might represent the immune-exclusive type and respond to immunotherapy only in case of immunity inducers, such as chemotherapy, radiation, or target therapy (Chen and Mellman, 2017). The third subtype was characterized by metabolism pathways and a lack of immune cells, thus representing the immune-ignored tumors, which might not benefit from immunotherapy and should be treated with cytotoxic and targeting medicines (Chen and Mellman, 2017). These results provide information for personalized therapy.

Besides subtyping, a scoring system to describe CRC features and guide treatment is also an interesting issue. For example, the immunoscore is a prognostic marker in CRC based on quantifying the lymphocyte populations at tumor centers and invasive margins (Bruni et al., 2020). This score correlates with neoantigen load, WNT/β-catenin signaling pathways, gut microbiota, and, most importantly, response to ICIs (Angell et al., 2020). Our m^6^Ascore also has a similar ability. A low m^6^Ascore indicated defected DNA response, high CD8^+^ T cells, low stromal activity, high mutation burdens, and prolonged survival. Although we do not have CRC cohorts treated with ICI, in two cohorts beyond CRC, the m^6^Ascore showed a prognostic value in terms of objective response, progression-free survival, and overall survival. Our m^6^Ascore might be used for clinical decisions as immunoscore, MSI, or RAS mutation. Signatures that predict the immune status or response to immunotherapy were also found in previous studies for CRC. For example, a STING-related prognostic score (Chen et al., 2021b) or an immune-related gene signature (Zhang et al., 2020b) has been shown to provide insights into immunotherapy. They were derived from existing gene pools. Unlike them, our m^6^Ascore was derived from m^6^A modulator–related genes. Some studies also utilized m^6^A regulators for signature construction. Zhang et al. (2020c) used two m6A readers, YTHDC2 and IGF2BP3, to construct a prognostic model in CRC. Jiang et al. (2021) found that an m^6^A-related lncRNA-based signature was associated with tumor-infiltrating immune cells. Chong et al. (2021) used a similar method to ours to construct an m^6^Ascore, but their study was confined to colon cancer and used fewer cohorts. These studies support our result of a close link between m^6^A and immune TME. Unfortunately, all these signatures were only studied by association to indirect factors favoring immunotherapy, but none were validated in the CRC-ICI cohorts.

This study also has several limitations. First, the m^6^A regulators were based on known genes with functions related to m^6^A modification. Clustering with a more comprehensive range of m^6^A regulators or signatures may result in better clinical values. Second, the subtyping and scoring systems were based on transcriptomic data. Methods based on PCR or immunostaining would be more feasible in clinical practice. Third, this study is retrospectively based on published cohorts. A prospective study is needed for medical translation in the future. Last, because there were no transcriptomic data from a CRC cohort with ICI treatment, we used two non-CRC cohorts for validation of m^6^Ascore in predicting responses to immunotherapy. Such an investigation in CRC patients would be of greater value.

In conclusion, we established a connection between m^6^A modification and the TME status in CRC. The m^6^A-based subtyping and scoring systems stratified CRC patients with different tumor immunity, molecular features, and clinical outcomes and have potential clinical implications in clinical decisions.

## Data Availability

The datasets presented in this study can be found in online repositories. The names of the repository/repositories and accession number(s) can be found in the article/Supplementary Material.
