# Supplementary material for: Links Between N 6-Methyladenosine and Tumor Microenvironments in Colorectal Cancer
Source: Front Cell Dev Biol. 2022 Feb 10;10:807129. doi: 10.3389/fcell.2022.807129 (PMC8866562; doi:10.3389/fcell.2022.807129)
Supplement: Supplementary file 1 [file DataSheet1.PDF]

Supplementary Material

1 Supplementary Figures

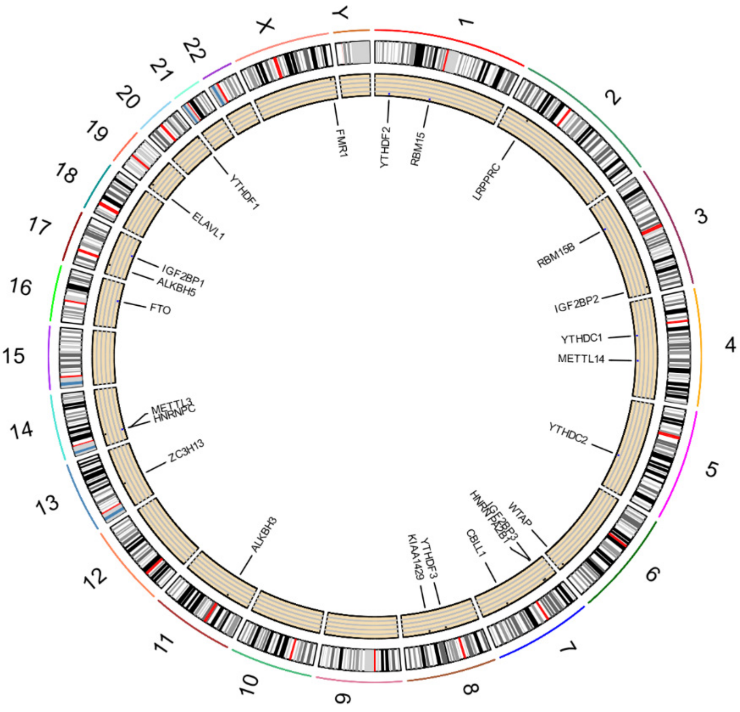

Supplementary Figure 1. Copy number alternations in CRC chromosomes and location of m^{6}A regulators.

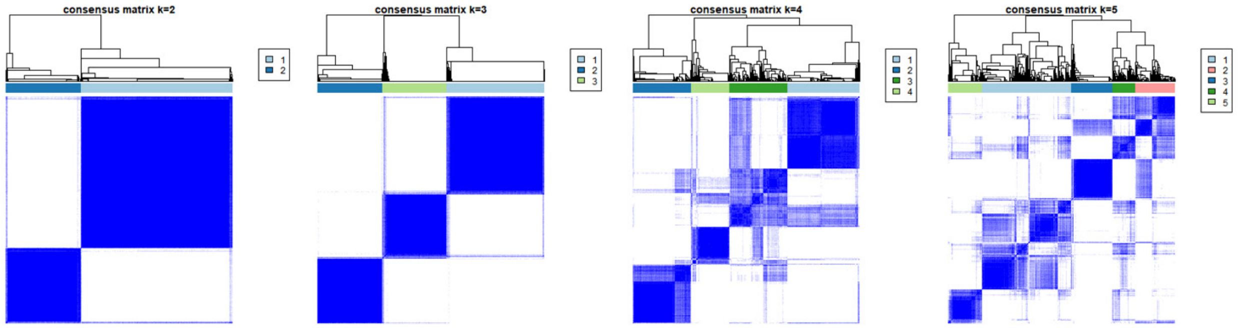

Supplementary Figure 2. Unsupervised clustering based on m^{6}A regulators with n = 2 to 5 in the meta-GEO dataset.

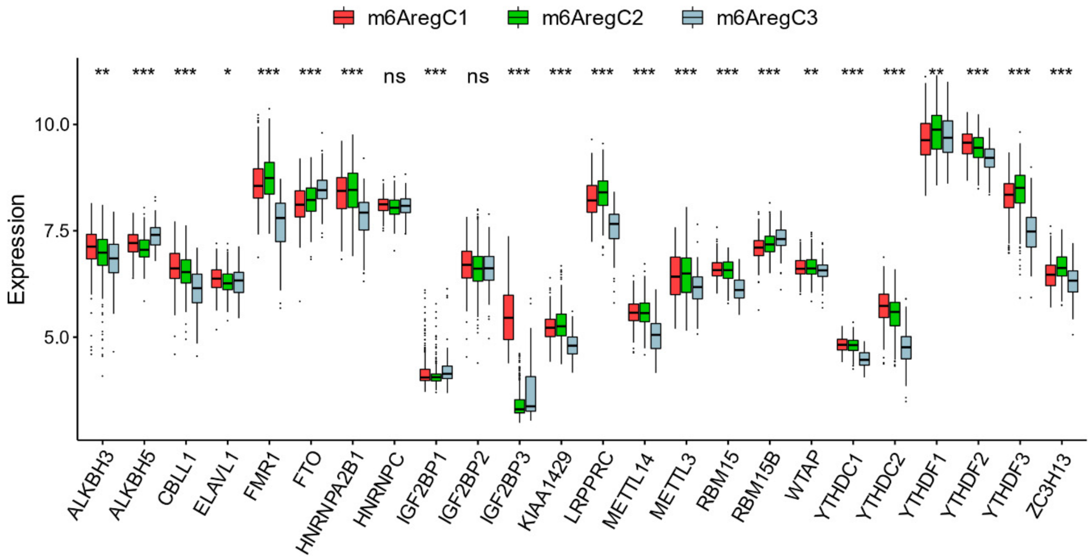

Supplementary Figure 3. Expression of m6A regulators in three m6A regulator-based clusters.

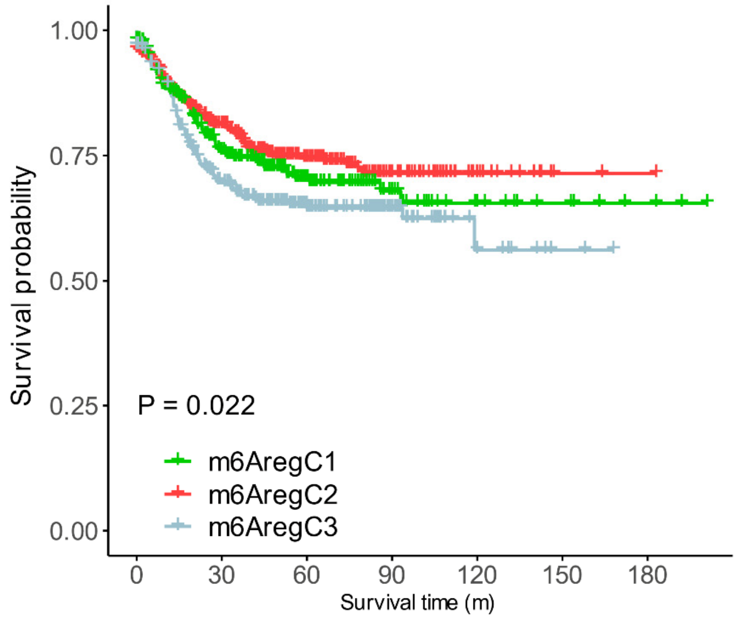

Supplementary Figure 4. Kaplan-Meier curves of the three m^{6}A regulator-based subtypes in the meta-GEO cohort.

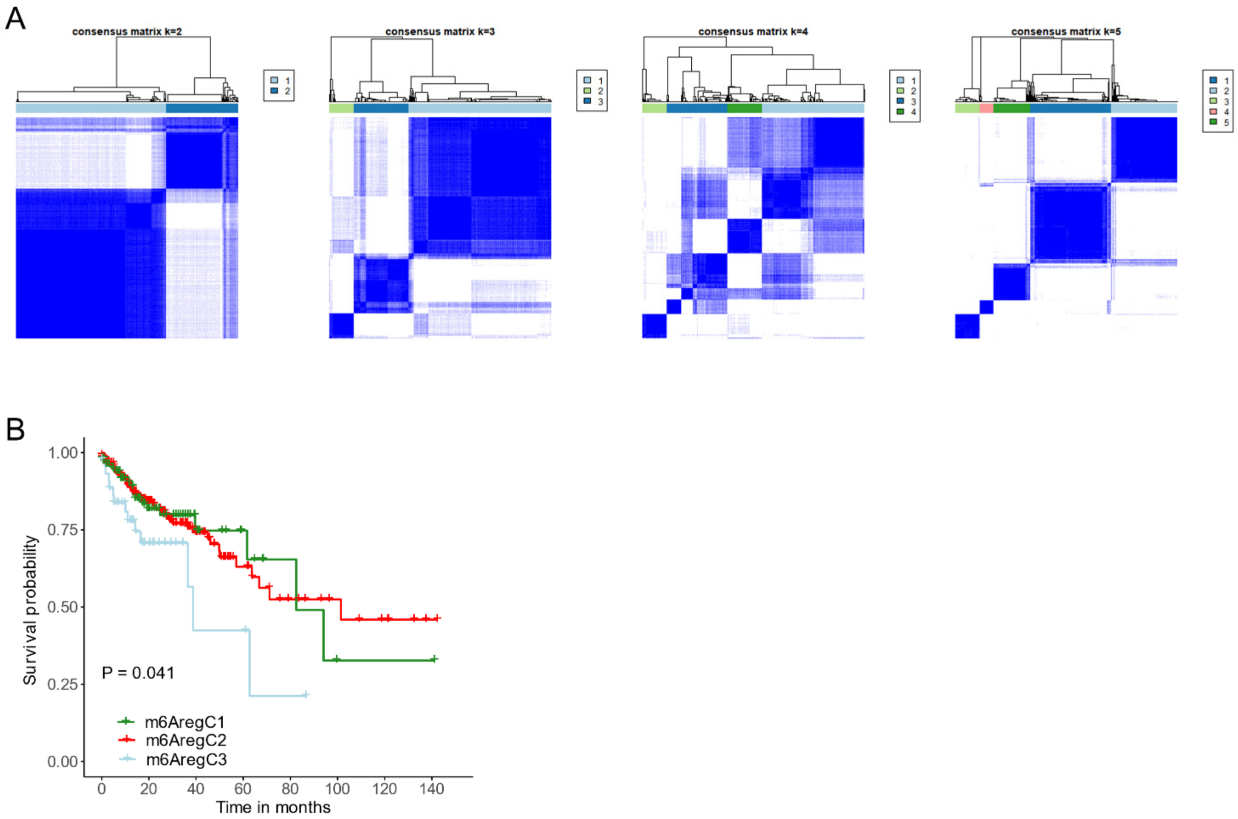

Supplementary Figure 5. Unsupervised clustering and survival differences in the TCGA dataset. A. Unsupervised clustering based on m^{6}A regulators with n = 2 to 5 in the TCGA dataset. B. Kaplan-Meier curves of the three m6A regulator-based subtypes in the TCGA dataset.

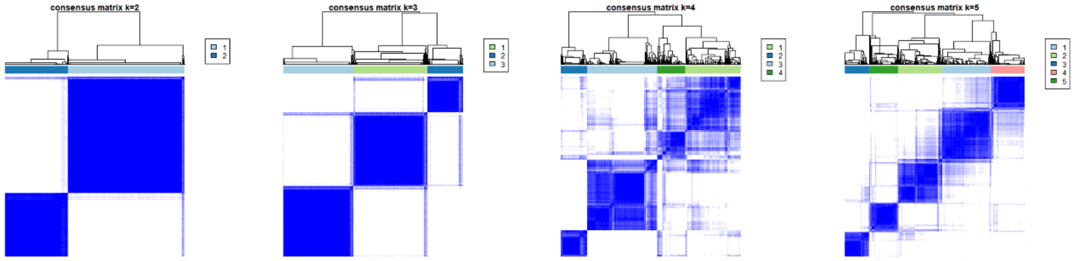

Supplementary Figure 6. Unsupervised clustering based on m^{6}A signatures with n = 2 to 5 in the GSE39582 dataset.
